# Autosomal Recessive Bestrophinopathy: Clinical Features, Natural History, and Genetic Findings in Preparation for Clinical Trials

**DOI:** 10.1016/j.ophtha.2020.10.006

**Published:** 2021-05

**Authors:** Giuseppe Casalino, Kamron N. Khan, Monica Armengol, Genevieve Wright, Nikolas Pontikos, Michalis Georgiou, Andrew R. Webster, Anthony G. Robson, Parampal S. Grewal, Michel Michaelides

**Affiliations:** 1 Moorfields Eye Hospital NHS Foundation Trust, and UCL Institute of Ophthalmology, University College London, London, United Kingdom; 2 Oftalmico Hospital, ASST Fatebenefratelli Sacco, Milan, Italy; 3 Leeds Teaching Hospitals NHS Trust, Leeds, United Kingdom; 4 Guy’s and St. Thomas’ Hospital NHS Foundation Trust, London, United Kingdom

**Keywords:** Autosomal recessive bestrophinopathy, *BEST1*, Electrophysiology, Gene therapy, Genetics, Retinal imaging, ADB, autosomal dominant Best disease, ARB, autosomal recessive bestrophinopathy, CNV, choroidal neovascularization, CRT, central retinal thickness, DA, dark-adapted, FAF, fundus autofluorescence, FCE, focal choroidal excavation, IRF, intraretinal fluid, LA, light-adapted, logMAR, logarithm of the minimum angle of resolution, NIR, near-infrared reflectance, RPE, retinal pigment epithelium, SRF, subretinal fluid, VA, visual acuity

## Abstract

**Purpose:**

To investigate the clinical course, genetic findings, and phenotypic spectrum of autosomal recessive bestrophinopathy (ARB) in a large cohort of children and adults.

**Design:**

Retrospective case series.

**Participants:**

Patients with a detailed clinical phenotype consistent with ARB, biallelic likely disease-causing sequence variants in the *BEST1* gene, or both identified at a single tertiary referral center.

**Methods:**

Review of case notes, retinal imaging (color fundus photography, fundus autofluorescence, OCT), electrophysiologic assessment, and molecular genetic testing.

**Main Outcome Measures:**

Visual acuity (VA), retinal imaging, and electrophysiologic changes over time.

**Results:**

Fifty-six eyes of 28 unrelated patients were included. Compound heterozygous variants were detected in most patients (19/27), with 6 alleles recurring in apparently unrelated individuals, the most common of which was c.422G→A, p.(Arg141His; n = 4 patients). Mean presenting VA was 0.52 ± 0.36 logarithm of the minimum angle of resolution (logMAR), and final VA was 0.81 ± 0.75 logMAR (*P* = 0.06). The mean rate of change in VA was 0.05 ± 0.13 logMAR/year. A significant change in VA was detected in patients with a follow-up of 5 years or more (n = 18) compared with patients with a follow-up of 5 years or less (n = 10; *P* = 0.001). Presence of subretinal fluid and vitelliform material were early findings in most patients, and this did not change substantially over time. A reduction in central retinal thickness was detected in most eyes (80.4%) over the course of follow-up. Many patients (10/26) showed evidence of generalized rod and cone system dysfunction. These patients were older (*P* < 0.001) and had worse VA (*P* = 0.02) than those with normal full-field electroretinography results.

**Conclusions:**

Although patients with ARB are presumed to have no functioning bestrophin channels, significant phenotypic heterogeneity is evident. The clinical course is characterized by a progressive loss of vision with a slow rate of decline, providing a wide therapeutic window for anticipated future treatment strategies.

The bestrophinopathies are a spectrum of inherited retinal dystrophies caused by pathogenic variation in the Bestrophin1 protein, encoded by the *BEST1* gene.^1^^,^^2^ The gene product is a pentameric calcium-sensitive chloride channel that localizes to the basolateral plasma membrane of the retinal pigment epithelium (RPE).^2^, ^3^, ^4^ The channel regulates the flow of chloride and other anions based on intracellular calcium concentrations. Recent studies have improved our understanding of the architecture and function of this channel, consisting of a central ion pore and calcium-dependent gating apparatus. Pathogenic mutations are prevalent in the gating apparatus.^5^^,^^6^

A wide array of unique *BEST1* variants have been reported, advancing our understanding of how genotypes influence phenotypes. The most prevalent variants are transmitted in an autosomal dominant pattern and are found in the first half of the gene, predicted to result in heterozygous missense variants.^1^^,^^2^ *BEST1* haploinsufficiency seems to be tolerated, suggesting that dominant mutations act by conferring a gain-of-function effect; however, this remains controversial.^7^ Phenotypes associated with heterozygous pathogenic variants include: (1) conditions that predominantly affect the macula, including Best disease (Online Mendelian Inheritance in Man identifier, 153700) and adult vitelliform macular dystrophy (Online Mendelian Inheritance in Man identifier, 153840); (2) those with generalized retinal involvement, including autosomal dominant vitreoretinochoroidopathy and rod–cone dystrophy; and (3) diseases with retinal and anterior segment involvement, including autosomal dominant microcornea, rod–cone dystrophy, early-onset cataract, and posterior staphyloma.^1^

In 2006, Schatz et al^8^ were the first to report 2 related patients harboring compound *BEST1* heterozygous variants and demonstrating a multifocal vitelliform dystrophy. Two years later, Burgess et al^9^ concluded that this condition was a fourth *BEST1*-associated phenotype and coined the term *autosomal recessive bestrophinopathy* (ARB). The clinical features of ARB include multifocal vitelliform deposits and irregularity of the RPE, evident as hyperautofluorescent and hypoautofluorescent areas at the posterior pole (Fig 1), intraretinal fluid (IRF), hypermetropia, and in some, shallow anterior chambers, predisposing them to angle-closure glaucoma.^9^ The electro-oculogram light peak-to-dark trough ratio usually is severely reduced because of severe generalized RPE dysfunction. Full-field electroretinography typically shows abnormal results from late childhood or adolescence and indicates generalized rod and cone dysfunction; however, this is insufficient to explain the severe electro-oculography reduction. In addition, pattern electroretinography evidence of macular dysfunction is found.^9^

**Figure 1:**
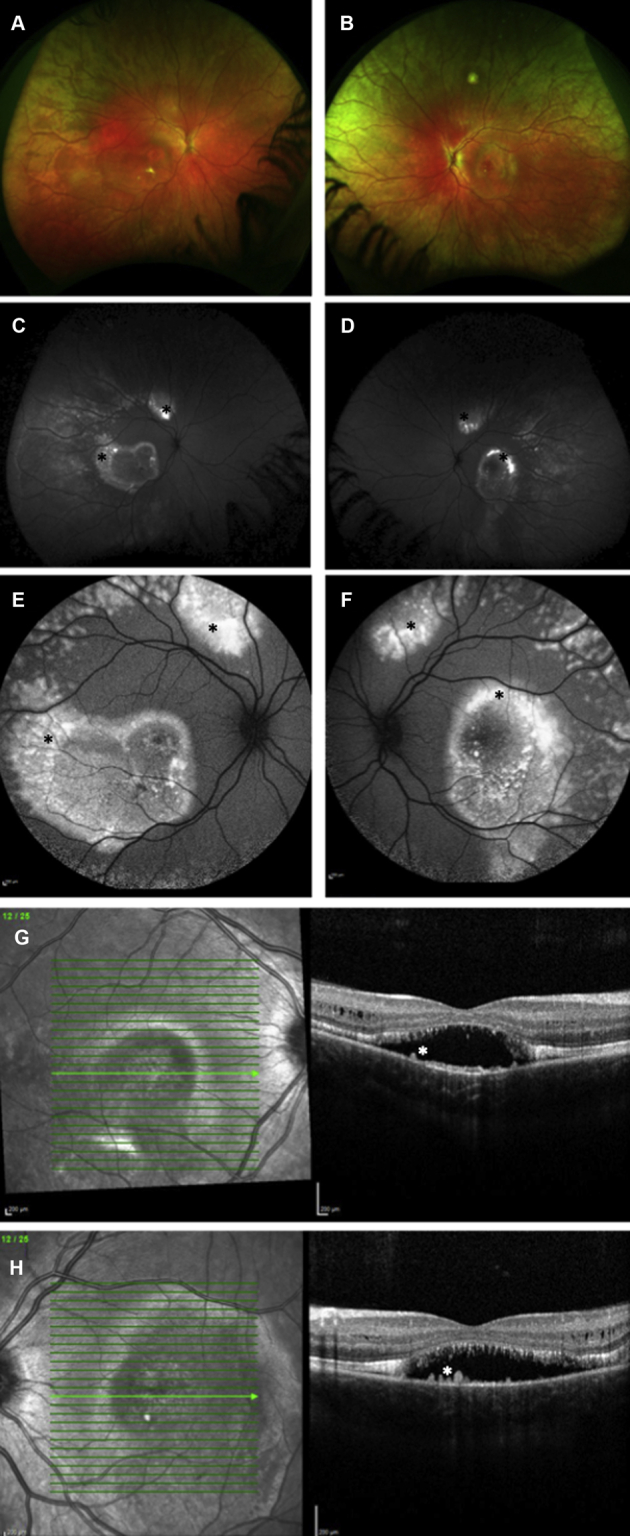
Multimodal retinal imaging of patient 22 (p.Tyr97Ter and p.Leu41Pro mutations in *BEST1*). **A**, **B**, Widefield color images showing multifocal vitelliform material (VM) in both eyes. **C**, **D**, Widefield fundus autofluorescence and (**E**, **F**), fundus autofluorescence (55°) images showing marked increased autofluorescence in correspondence to the VM areas (black asterisks). **G**, **H**, Spectral-domain OCT scan of both eyes showing subretinal drusen-like deposits (white asterisks), subretinal fluid, outer retinal layer thickening, and intraretinal fluid.

Currently, considerable interest exists in developing therapy for patients with inherited retinal disease, with gene replacement being the most advanced strategy at present. Voretigene neparvovec-rzyl (Luxturna, Spark Therapeutics) already is available for the treatment of biallelic *RPE65*-associated retinal dystrophy, with further trials underway to treat *CHM-*, *RS1*-, *RPGR-*, *MERTK*-, *ABCA4*-, *USH2A-*, *MY07A-*, *CNGA3-*, and *CNGB3*-associated retinal disease.^10^ Autosomal recessive bestrophinopathy conceivably should be amenable to a similar therapeutic approach, and a recent study using gene therapy to treat the canine model of *BEST1*-associated retinopathy confirmed this.^11^ The current study provides a detailed characterization of the clinical phenotype, genetic findings, and the natural history of ARB in a large number of patients from a single institution, aiming to assist the design of anticipated clinical therapeutic trials for this disease and to help inform advice on prognosis.

## Methods

### Patient Identification and Assessment

Clinical records and multimodal retinal imaging of patients with ARB attending a tertiary referral center, Moorfields Eye Hospital in London, United Kingdom, were reviewed.^12^ Patients known to the eye clinic with a diagnosis of ARB were identified using in-house databases (OpenEyes, London, United Kingdom). Electronic healthcare records and case notes then were reviewed. All patients included in this database had provided informed consent. This retrospective study adhered to the tenets of the Declaration of Helsinki and was approved by the Moorfields Eye Hospital ethics committee.

Clinical notes, retinal imaging, and visual electrophysiologic features were reviewed. Patient ethnicity was recorded according to the United States Department of Health and Human Services (https://ushik.ahrq.gov). Clinical data extracted included visual acuity (VA), refraction, slit-lamp biomicroscopy, and funduscopy findings. Color fundus photography, near infrared reflectance (NIR) imaging, OCT, and fundus autofluorescence (FAF) imaging were reviewed for all patients. On the basis of the age of onset, we distinguished between patients with adult-onset (>18 years of age) and childhood-onset (<18 years of age) disease.

Visual acuity data at first visit (presentation) and at the most recent follow-up visit (final) were analyzed. Where necessary, Snellen acuity was converted into logarithm of the minimum angle of resolution (logMAR). Color fundus photography was obtained with either the Optos widefield camera (Optos Panoramic 200; Optos PLC) or the TRC-50LA retinal fundus camera (Topcon). Near-infrared reflectance and OCT imaging were performed simultaneously using the Spectralis SD-OCT device (Heidelberg Engineering) for all patients. Fundus autofluorescence images were obtained with either a Spectralis HRA OCT (Heidelberg Engineering) or Optos widefield camera (Optos PLC). When necessary, fluorescein angiography was performed on either the Spectralis or retinal fundus camera. Visual electrophysiologic testing incorporated the International Society of Clinical Electrophysiology of Vision standards and included electro-oculography, dark-adapted (DA) and light-adapted (LA) full-field electroretinography, and pattern electroretinography.^13^, ^14^, ^15^ Change in full-field electroretinography response over time was assessed by comparing results obtained from patients with ARB with those from unaffected, age-matched control individuals (in-house database, n = 140).

### Imaging Grading

Multimodal imaging, including NIR, OCT, FAF, and color fundus photography, at presentation and most recent follow-up visit were reviewed. OCT analysis included grading for presence of drusen-like vitelliform material (defined as accumulation of subretinal deposits with a hyperreflective appearance on tomographic scan),^16^ outer retinal layer thickening (defined as a thicker layer between the RPE and ellipsoid zone interface^17^ corresponding to the interdigitation zone according to the consensus of definitions of OCT nomenclature),^18^ the presence of IRF (defined qualitatively as >3 adjacent intraretinal hyporeflective spaces visible on OCT), pigment epithelial detachment (defined as separation between the RPE and Bruch’s membrane), and subretinal fluid (SRF). The presence of SRF was categorized further as either diffuse (throughout the entire line scan passing through the fovea) or focal (subfoveal fluid only).

Presence of macular RPE atrophy and macular fibrosis also were assessed. Macular RPE atrophy was defined as single or multiple confluent areas of hyperreflectivity with sharp margins on NIR and visible large choroidal vessels on fundus photographs that corresponded to choroidal signal enhancement with loss of RPE and choroidal hypertransmission on the accompanying OCT scans.^19^ Macular fibrosis identification was based on fundus photograph, NIR, and OCT characteristics. On fundus photographs, fibrosis was said to be present if well-delineated areas of yellow-white tissue with corresponding increased reflectivity were present on NIR and well-defined hyperreflective material was present on the accompanying OCT images.^19^ Central retinal thickness (CRT) from the central 1-mm subfield was determined using the Heidelberg software, after manual inspection to ensure correct centration and segmentation. The presence of focal choroidal excavation (FCE)^20^ and choroidal neovascularization^21^^,^^22^ also were investigated. Choroidal neovascularization was identified on the basis of fluorescein angiography findings. The nature of material deposited in the subretinal space also was evaluated. Subretinal deposit was defined as subretinal yellowish material on color fundus photography, with corresponding hyperreflective material on OCT and increased autofluorescence on FAF and was classified as either unifocal or multifocal; subfoveal involvement also was assessed. The label of vitelliform material was reserved for significant collections of coalesced subretinal deposit, such that they resembled the yolk of an egg (Latin, *vitellus*), as typically observed in patients with autosomal dominant Best disease. All patients were evaluated for the presence of an inferior track sign on FAF indicating presumed gravitational tracking of SRF (chronicity). Where available, Optos widefield images were graded for presence of peripheral drusen-like material, defined as the accumulation of subretinal deposits without decreased FAF signal and the presence of RPE atrophy, defined as visible large choroidal vessels with corresponding decreased FAF signal.

### Molecular Diagnosis

Molecular genetic testing was as part of routine National Health Service care using single-gene Sanger sequencing or targeted capture next-generation sequencing (National Genetics Reference Laboratory, Manchester Centre for Genomic Medicine, Manchester, United Kingdom, and Molecular Vision Laboratory, https://www.molecularvisionlab.com/). Some alleles initially were found as part of whole-genome sequencing research projects (NIHR BioResource Rare Diseases Study and the Genomics England study).^23^^,^^24^ Segregation studies were performed where possible to confirm heterozygous variants were in trans in the affected probands. The nucleotide and peptide variants reported herein refer to transcript ENSTxxx and peptide ENSPxxx, respectively.

### Statistical Analysis

Data were analyzed using the Statistical Packages for Social Sciences software version 22 (IBM Corp). Descriptive statistics were generated for continuous variables and categorical variables. Statistical analysis largely was descriptive, except for the change in VA, which was converted from Snellen into logMAR units. Analysis of variance for nonparametric data distribution was used to study the differences in the VA between groups of patients based on the age at the time of diagnosis and on the length of follow-up. For statistical purposes only, VA in the right eye was considered for each patient. A cross-sectional analysis was performed for the electrophysiologic findings. The chosen level of statistical significance was *P* < 0.05.

## Results

Fifty-six eyes of 28 unrelated patients were included. Characteristics of patients are summarized in Table 1. At the time of initial examination, the mean age of the cohort was 26.7 ± 15.3 years (range, 4–63 years), and 10 patients were 18 years of age or younger (childhood-onset disease). Thirteen patients were female. Refractive correction was recorded for 15 patients, with all but 1 patient being hyperopic (Table 1). Eight patients demonstrated angle-closure glaucoma; 5 of these patients underwent bilateral peripheral laser iridotomy, and 4 of these patients underwent bilateral clear lens extraction. The most common presenting symptom was reduced central vision (12/18), with a minority of patients demonstrating acute angle-closure glaucoma (2/28), strabismus (2/18), or as an incidental finding on routine examination (2/18). Presenting symptoms were not available on review of case notes for 10 of 28 patients.

**Table 1:** Demographic Characteristics, Refraction, Visual Acuity, Presence of Glaucoma, and Years of Follow-up of Included Patients

Patient No.	Gender	Race	Age (yrs)	Refraction		Visual Acuity at First Visit∗		Visual Acuity at Last Visit∗		Primary Angle-Closure Glaucoma	Follow-up (yrs)
				Right Eye	Left Eye	Right Eye	Left Eye	Right Eye	Left Eye		
1	F	White	34	Unknown	Unknown	0.30	0.78	3.00	0.82	Yes	17
2	M	White	39	+4.00	+4/–0.50@70	1.00	1.00	1.00	1.00	Yes	14
3	M	Asian	49	+2/–0.50@120	+2/–0.75@70	0.78	0.78	0.78	0.78	No	15
4	M	White	30	Unknown	Unknown	0.60	0.60	0.60	0.60	No	11
5	M	Asian	22	Unknown	Unknown	1.00	1.00	1.00	1.00	No	12
6	F	White	44	+2.50	+4.00	0.60	0.48	0.90	0.90	No	15
7	F	White	27	+4.00	+4.00	0.78	1.00	0.60	1.30	No	18
8	M	White	48	Unknown	Unknown	0.48	0.48	1.60	1.60	No	14
9	M	White	36	Unknown	Unknown	0.48	1.00	0.48	0.78	No	7
10	F	Asian	35	Unknown	Unknown	1.00	0.78	1.60	1.60	Yes	12
11	F	Unknown	16	+1.25/+1@75	+1.00	0.18	0.48	0.18	0.48	Yes	12
12	F	White	5	+6/–1@180	+5/–0.75@50	0.10	0.10	0.90	0.20	No	11
13	M	Black	19	Unknown	Unknown	0.48	0.30	1.00	0.8	No	10
14	M	White	4	+4.50	+3.50	0.10	0.10	0.10	0.00	No	11
15	M	White	14	Unknown	Unknown	0.40	0.78	1.00	1.00	No	9
16	M	Unknown	11	+6/–0.75@110	+5.25/–1@80	0.32	0.02	0.18	0.00	No	8
17	M	Asian	40	Unknown	Unknown	0.60	0.30	0.60	0.60	Yes	9
18	M	Unknown	12	–0.50	–0.25	0.06	0.40	0.00	0.00	No	8
19	F	White	15	+4.00	+3.00	0.56	0.10	0.42	0.02	No	2
20	M	White	45	Unknown	Unknown	1.60	1.60	3.00	3.00	No	3
21	F	White	25	Unknown	Unknown	0.78	1.18	1.00	1.60	Yes	4
22	F	White	12	+0.50	+0.75/–0.25@5	0.30	0.28	0.48	0.48	No	4
23	M	Black	12	+1.00	+0.25	0.30	0.28	0.18	0.30	No	3
24	M	Black	31	–1.00	–1.25	0.18	0.18	0.30	0.18	No	1
25	F	White	63	Unknown	Unknown	0.30	0.78	0.30	0.60	No	3
26	F	White	31	+2.50	+3.00	0.30	0.18	0.18	0.18	Yes	2
27	F	Asian	7	+5/–1@180	+6/–2@180	0.12	0.20	0.40	0.50	No	1
28	F	White	22	Unknown	Unknown	0.78	0.30	1.00	0.48	Yes	2

F = female; M = male.

∗ In logarithm of the minimum angle of resolution units.

### Visual Acuity Progression

**Figure 2:**
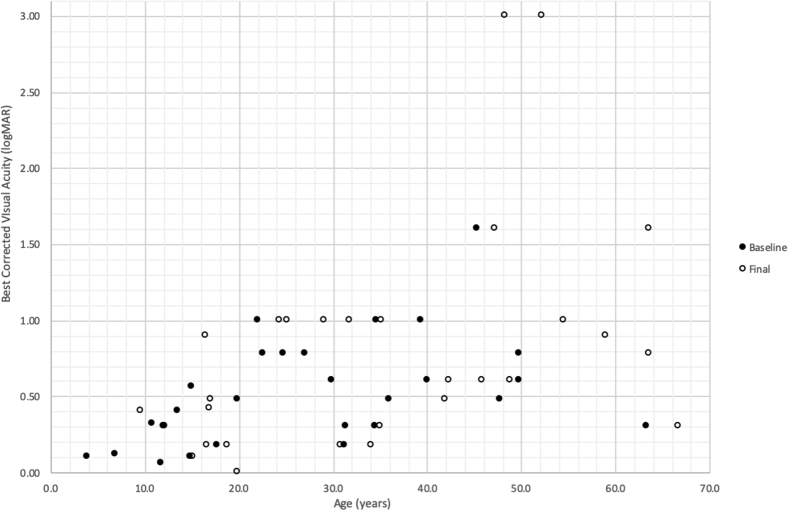
Scatterplot depicting best-corrected visual acuity (logarithm of the minimum angle of resolution units) as a function of age (years). Vision for the right eye at baseline (on first presentation to our facility) and at final follow-up is depicted for each patient as per the legend.

Between initial and final assessments, VA declined in most patients (80.4%; mean follow-up, 8.6 ± 5.3 years; range, 1.7–18.8 years). A significant change in VA was detected in patients with 5 years or more of follow-up (n = 18) compared with patients with 5 years or less of follow-up (n = 10; *P* = 0.001). As a group, the mean presenting VA was 0.52 ± 0.36 logMAR, and final VA was 0.81 ± 0.75 logMAR (*P* = 0.06). Younger patients (those ≤18 years of age) recorded better acuity compared with older patients (*P* < 0.001). The mean rate of VA decline for children (<18 years of age) was 0.05 ± 0.16 logMAR/year, the same as for adults, 0.05 ± 0.12 logMAR/year (*P* = 1.00). The mean rate of change in VA was 0.05 ± 0.13 logMAR/year. Right and left eyes did not differ in mean presenting VA (0.55 ± 0.40 logMAR; *P* = 0.40), mean final VA (0.74 ± 0.65 logMAR; *P* = 0.65), or mean rate of change in VA (0.04 ± 0.10 logMAR; *P* = 0.10). Further subgroup analysis was conducted based on presenting VA. This was divided into group 1 (VA, ≤0.3 logMAR), group 2 (VA, >0.3 and ≤0.6 logMAR), and group 3 (VA, >0.6 logMAR). Group 1 showed a mean progression of 0.15 ± 0.15 logMAR/year (11 eyes). Group 2 showed a mean progression of 0.04 ± 0.04 logMAR/year (9 eyes; *P* = 0.30). Group 3 showed a mean progression of 0.09 ± 0.17 logMAR/year (8 eyes; *P* = 0.78). Figure 2 depicts a scatterplot including the presenting and final BCVA for each patient.

### Molecular Genetic Findings

Biallelic disease-causing variants were identified in each of 27 simplex probands from 27 unrelated families. One patient (patient 19) demonstrated typical clinical, imaging, and electroretinography phenotype of ARB but declined molecular testing (Table 2). Of the 27 patients who did undergo genetic screening, 8 were homozygous and 19 were compound heterozygotes for *BEST1* variants.

**Table 2:** List of Detected Variants in Enrolled Patients

Patient No.	Variant 1	Predicted Effect	Variant 2	Predicted Effect
1	c.102C→T	p.Gly34Gly	c.572T→C	p.Leu191Pro
2	c.102C→T	p.Gly34Gly	c.1470_1471delCA	p.His490GlnfsTer24
3	c.-29+1G→T	splicing	c.-29+1G→T	Splicing
4	*c.1014G*→*A*	p.Trp338Ter	c.-29+1G→T	Splicing
5	c.418C→G	p.Leu140Val	c.418C→G	p.Leu140Val
6	c.454C→G	p.Pro152Ala	c.122T→C	p.Leu41Pro
7	c.122T→C	p.Leu41Pro	c.422G→A	p.Arg141His
8	c.454C→G	p.Pro152Ala	c.584C→T	p.Ala195Val
9	c.598C→T	p.Arg200Ter	c.598C→T	p.Arg200Ter
10	c.107_118delAGTACGAGAACC	p.Gln36_Asn39del	c.107_118delAGTACGAGAACC	p.Gln36_Asn39del
11	c.1038dupC	p.Tyr347LeufsTer54	*c.533A*→*C*	p.His178Pro
12	c.422G→A	p.Arg141His	c.475C→T	p.Gln159Ter
13	c.636+1G→C	splicing	c.636+1G→C	Splicing
14	c.584C→T	p.Ala195Val	c.974T→C	p.Met325Thr
15	c.1066C→T	p.Arg356Ter	Exon 1 to 2 deletion	Not applicable
16	c.1066C→T	p.Arg356Ter	c.550C→T	p.Pro184Ser
17	c.468C→G	p.His156Gln	c.468C→G	p.His156Gln
18	c.1066C→T	p.Arg356Ter	c.602T→C	p.Ile201Thr
19	Declined genetic testing			
20	c.974T→C	p.Met325Thr	c.602T→C	p.Ile201Thr
21	c.29C→T	p.Ala10Val	c.422G→A	p.Arg141His
22	c.291C→G	p.Tyr97Ter	c.122T→C	p.Leu41Pro
23	c.74G→A	p.Arg25Gln	c.278G→A	p.Trp93Ter
24	c.530C→T	p.Pro177Leu	c.169G→T	p.Glu57Ter
25	c.1038dupC	p.Tyr347LeufsTer54	c.421C→A	p.Arg141Ser
26	c.728C→A	p.Ala243Glu	c.728C→A	p.Ala243Glu
27	c.418C→G	p.Leu140Val	c.418C→G	p.Leu140Val
28	c.422G→A	p.Arg141His	c.839A→C	p.Gln280Pro

In total, 31 unique, rare, likely disease-associated variants were reported on the 54 *BEST1* alleles of the 27 probands*.* These included 18 missense and 13 others predicted to be null alleles (9 protein-truncating variants, 2 mutations affecting a canonical splice donor site sequence, 1 in-frame deletion of 12 nucleotides [4 amino acids], and 1 multiexon deletion).

Biallelic missense variants were the most frequently detected combination of pathogenic alleles (12/27), followed by null and missense alleles (9/27), with 2 null alleles being identified in only a minority of patients (6/27). Pathogenic variants were detected in a homozygous state in 8 patients; 2 of these patients (patients 5 and 27) with the same ethnicity shared the same variant (c.418C→G, p.(Leu140Val)) without being knowingly related. Compound heterozygous variants were detected in the majority of patients (19/27), with 11 alleles recurring in apparently unrelated individuals, the most common of which was c.422G→A, p.(Arg141His), seen in 4 unrelated patients (patients 7, 12, 21, and 28). Novel variants were defined as absent from gnomAD version 2.1.1 (accessed June 18, 2020) and not published previously or reported in Clinvar. Nine novel variants were identified in our cohort, which included 5 novel missense and 4 novel protein-truncating variants (Table S1, available at www.aaojournal.org).

Comparing the pathogenicity score (Combined Annotation Dependent Deletion; Table S1) of our reported ARB missense variants (n = 18) with those reported in gnomAD (n = 397), we found it to be significantly higher in our ARB variants (*P* < 0.001), as expected. Next, we compared the distributions of the peptide coordinates of our ARB missense variants with those reported to be associated with the dominant form of the disease (ADB) in Clinvar (n = 31) and a set of presumed benign missense variants from gnomAD (n = 397). Although the distributions of peptide locations for gnomAD were relatively uniform, a notable difference was found in the distributions of ARB and ADB peptides with apparent clustering (Fig S1, available at www.aaojournal.org). Autosomal recessive bestrophinopathy mutations were particularly enriched in the helical domain (amino acid positions 179–199) compared with gnomAD (Fig S1).

### Imaging Findings

**Figure 3:**
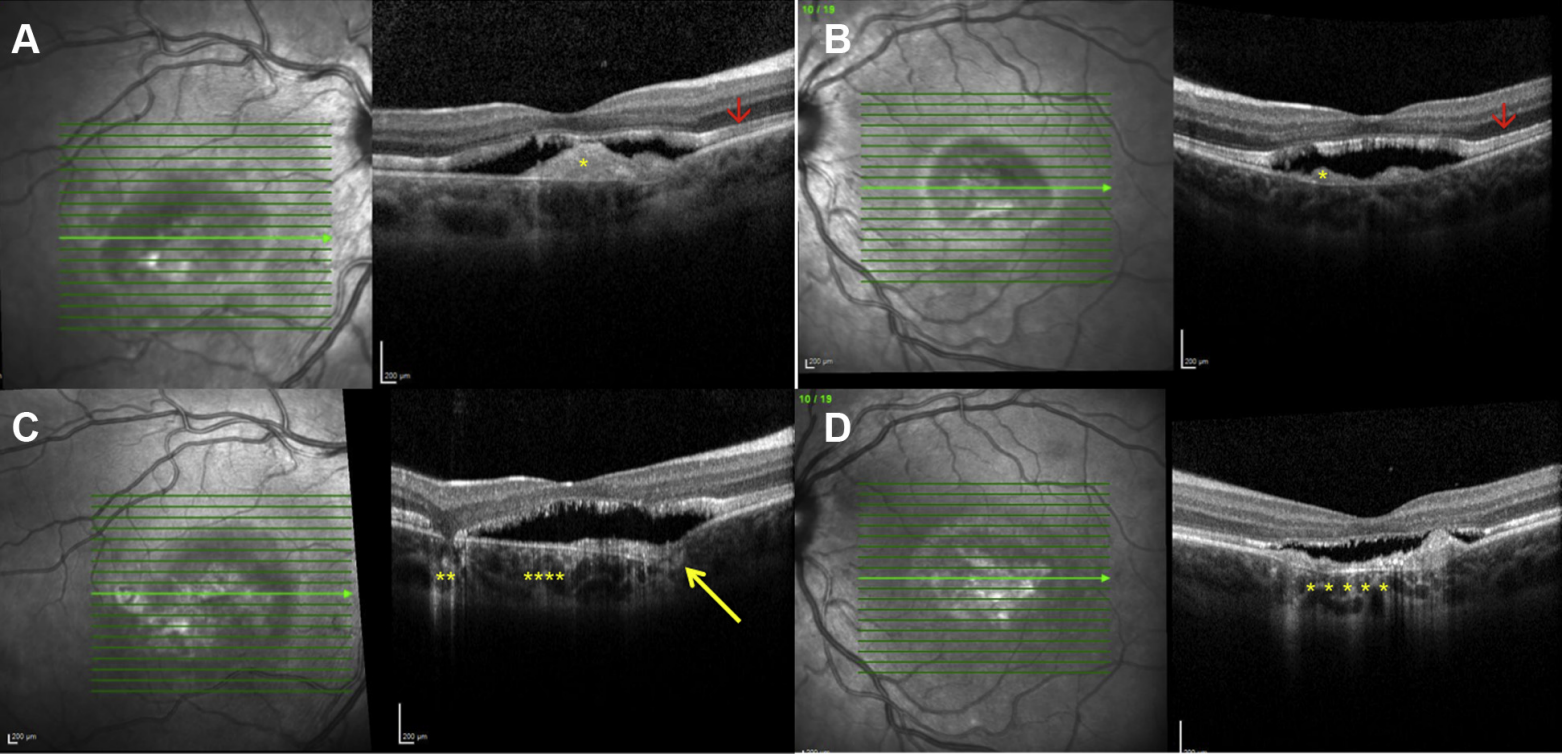
Spectral-domain OCT image from patient 12 obtained (**A**, **B**) at presentation and (**C**, **D**) at last follow-up visit. **A**, **B**, Spectral-domain OCT image obtained at presentation showing well-defined subretinal hyperreflectivity consistent with vitelliform material (yellow asterisks), subretinal fluid, outer retinal layer thickening (red arrows), and elongation of the photoreceptor outer segments (stalactites). **C**, Spectral-domain OCT image of the right eye showing persistent subretinal fluid, a focal choroidal excavation (yellow arrow), and backscattering of the signal in the choroid consistent with retinal pigment epithelium (RPE) atrophy (yellow asterisks). **D**, Spectral-domain OCT image of the left eye showing persistence of subretinal fluid and RPE atrophy at the macula (yellow asterisks).

**Figure 4:**
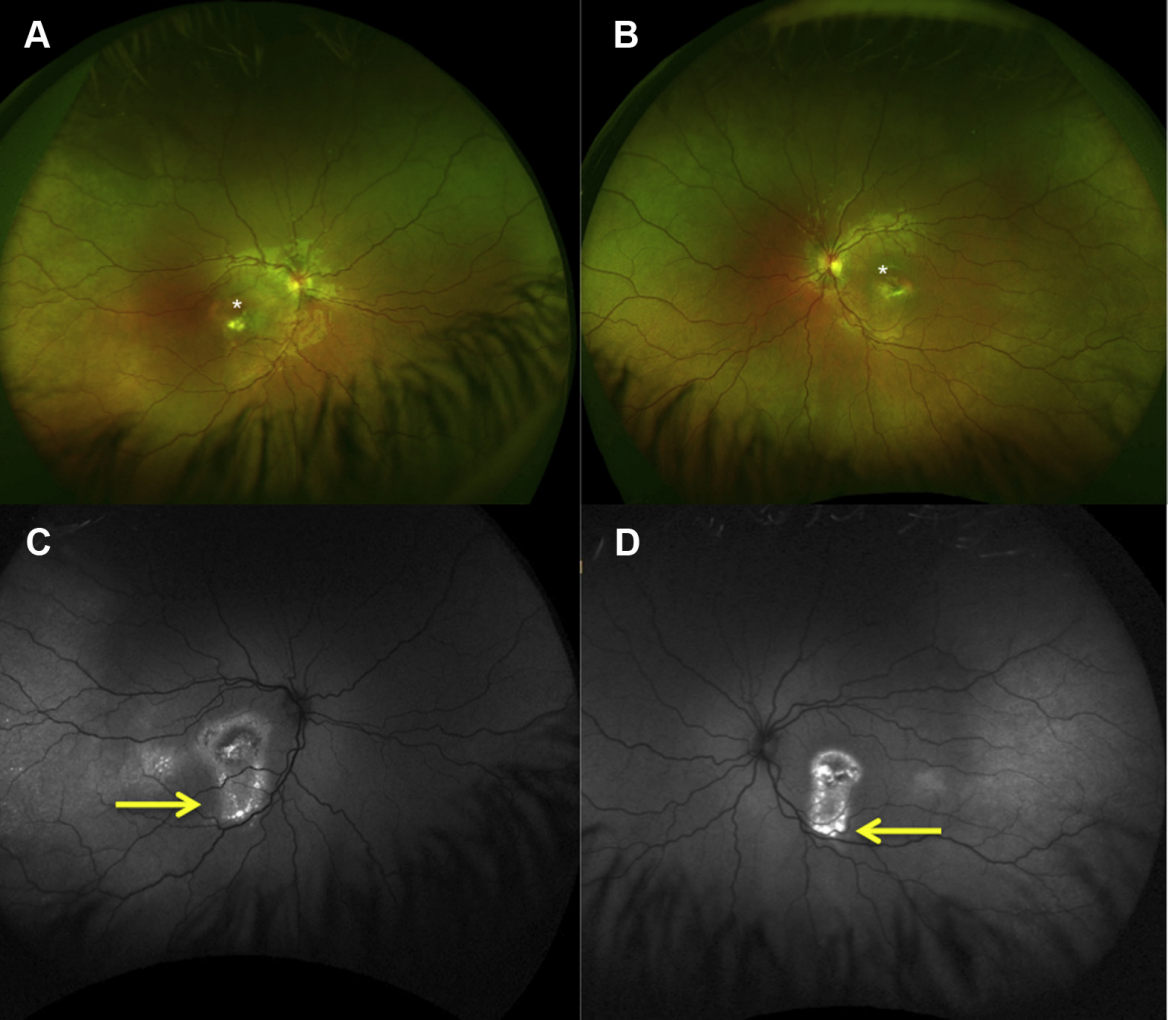
Optos widefield imaging from patient 12 (p.Arg141His and p.Gln159Ter mutations in *BEST1*). **A**, **B**, Widefield color images showing unifocal subfoveal vitelliform material in both eyes (white asterisks). **C**, **D**, Widefield fundus autofluorescence images showing marked increased autofluorescence at the posterior pole and increased autofluorescence (gravitational tract) tracking inferior to the macula (yellow arrows).

**Figure 5:**
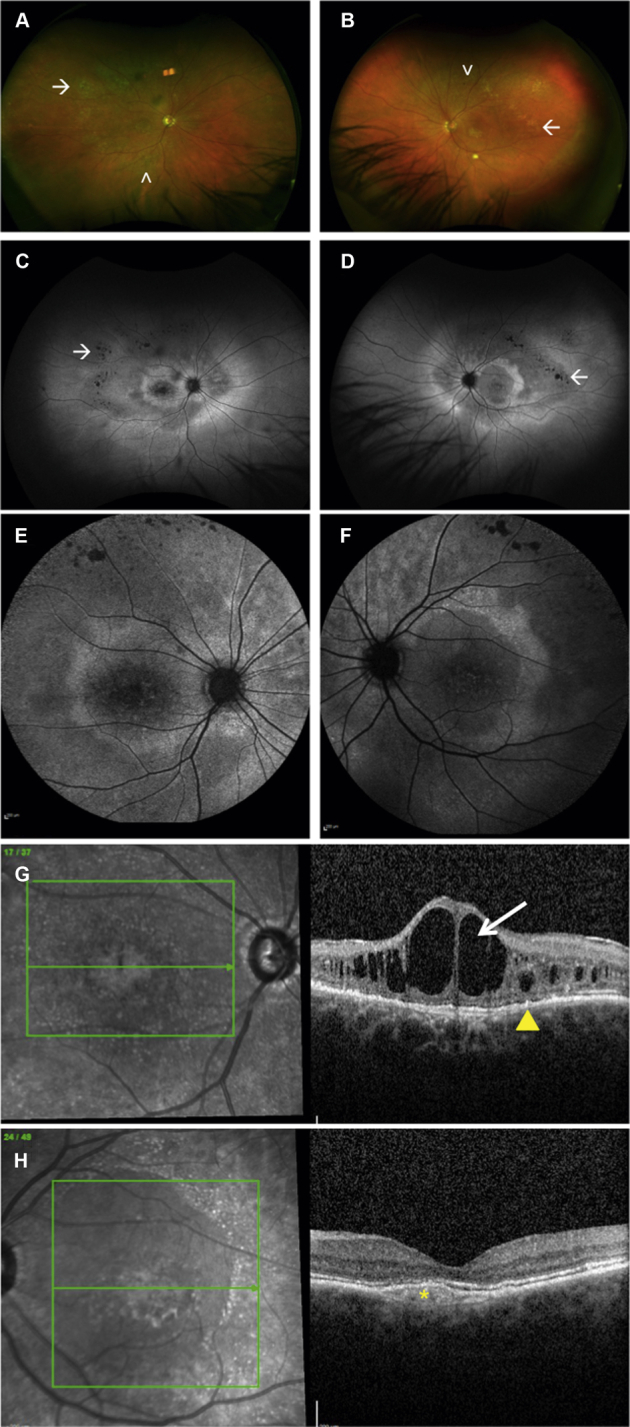
Multimodal retinal imaging from patient 1 (BEST1:p.Gly34Gly; p.Leu191Pro compound heterozygous). **A**, **B**, Widefield color images showing peripheral drusen-like material (white arrowheads) and patches of retinal pigment epithelium (RPE) atrophy in the periphery of both eyes (white arrows). **C**, **D**, Widefield fundus autofluorescence images showing marked autofluorescence changes at the posterior pole and in the mid periphery and decreased autofluorescence signal on correspondence of patches of RPE atrophy (white arrows). **E**, **F**, Fundus autofluorescence images (55°). **G**, Spectral-domain OCT images of the right eye showing cystoid macular degeneration (white arrow) and subretinal drusen-like deposits (yellow arrowhead). **H**, Spectral-domain OCT image of the left eye showing a shallow pigment epithelial detachment (yellow asterisk).

Retinal imaging analysis is presented in Table 3. Multimodal retinal imaging of patients 1, 12, and 22 are represented in Figures 1, 3, 4, and 5. Evidence of a high degree of interocular symmetry was found. The most prevalent imaging finding at presentation was subretinal deposit, which was found in most eyes (80.3% [45/56]) and most frequently was multifocal (69.6% [39/56]), with macular involvement in 17.85% of eyes (10/56). Prevalence of subretinal deposit did not increase over time. At final follow-up, a single vitelliform lesion, as is typically observed in patients with autosomal dominant Best disease, was present in 2 patients with ARB. Tomographic evidence of outer retinal layer thickening was identified in 46.4% of eyes (13/28) at both the initial and final examinations.

**Table 3:** Retinal Imaging Findings at Presentation and at Last Follow-up Visit

	Presentation	Last Follow-up
Spectralis OCT and FAF examination		
Macular SD		
Subfoveal	12 (6)	10 (5)
Unifocal	6 (3)	4 (2)
Multifocal	39 (20)	39 (20)
SRF		
Any	42 (22)	42 (22)
Subfoveal	20 (12)	22 (11)
Diffuse	22 (10)	22 (11)
IRF	32 (16)	34 (17)
ORL thickening	25 (13)	24 (12)
PED	3 (2)	4 (4)
FCE	3 (2)	8 (5)
Gravitational track	6 (6)	11 (6)
Macular RPE atrophy	11 (6)	13 (7)
Optos color and FAF examination		
Peripheral drusen-like material	19 (10)	
Peripheral atrophy	10 (6)	

FAF = fundus autofluorescence; FCE = focal choroidal excavation; IRF = intraretinal fluid; ORL = outer retina layer; PED = pigment epithelial detachment; RPE = retinal pigment epithelium; SD = subretinal deposit; SRF = subretinal fluid.

Subretinal fluid was found in most eyes (75% [42/56]) at presentation; the location of SRF was subfoveal in almost one half of these eyes and was diffuse (involving the entire OCT line scan) in the remaining patients. The presence of SRF did not change significantly over time, because it was found in the same proportion of eyes (75% [42/56]) at the last visit. More than one half of the eyes (57% [32/56]) demonstrated IRF at presentation, which remained relatively stable over time.

At presentation, macular RPE atrophy was identified in 39.2% of eyes (11/28), whereas macular fibrosis was found in a relatively small proportion of eyes (25% [7/28]). Retinal pigment epithelium atrophy and macular fibrosis were found in slightly more eyes at the last follow-up visit (Table 3).

Between initial and final OCT examinations, most patients (80.4%) recorded a reduction in CRT in the central 1-mm subfield. Mean CRT at baseline was 362 ± 139 μm (range, 147–754 μm) and at final follow-up was 349 ± 168 μm (range, 134–895 μm; *P* = 0.58). Subjectively, variation in CRT seemed to correlate with the degree of IRF, rather than outer retinal atrophy in this cohort of patients. Mean initial CRT in younger patients (≤18 years of age) was 403 ± 75 μm, whereas in adult patients, it was 339 ± 160 μm (*P* = 0.10). Most patients in both age groups (≤18 years, 90%; >18 years, 75%) showed a documented reduction in CRT at final follow-up.

During the initial examination, FCE was detected in 4 eyes of 3 patients, and at the last follow-up visit, it was detected in 8 eyes of 5 patients (Fig 3). In these eyes, FCE was not associated with evidence of type 1 macular neovascular disease; however, in 5 of 8 eyes, flat, irregular pigment epithelial detachments were present, associated with subretinal hyperreflective material in 3 patients, hinting that FCE may be associated with a relatively indolent type 2 neovascular lesion. One eye of 1 patient demonstrated a type 1 neovascular lesion without FCE (previously published) that did not require treatment.^17^^,^^18^

Changes in short-wavelength FAF were identified in all patients; hyperautofluorescence was observed in regions with outer retinal layer thickening, subretinal deposit, and subretinal fluid, and hyperautofluorescence was observed in regions of outer retinal atrophy. Gravitational tracks were noted in 6 eyes of 6 patients at the initial visit and in 11 eyes of 6 patients at the final visit (Fig 4). Qualitative longitudinal analysis identified an enlargement in the area of macular hypoautofluorescence in 14.3% of patients. For almost all patients, changes in FAF findings spared the peripapillary retina (26 of 28 [92.9%]).

Ultra-widefield imaging (Optos) was obtained in 42 eyes of 21 patients. Peripheral drusen-like material was visible in 19 eyes of 10 patients. Ten eyes of 6 patients manifested patches of peripheral RPE atrophy. All patients with peripheral atrophy showed evidence of peripheral (presumed subretinal) drusen-like material (Fig 5).

### Electrophysiologic Findings

**Figure 6:**
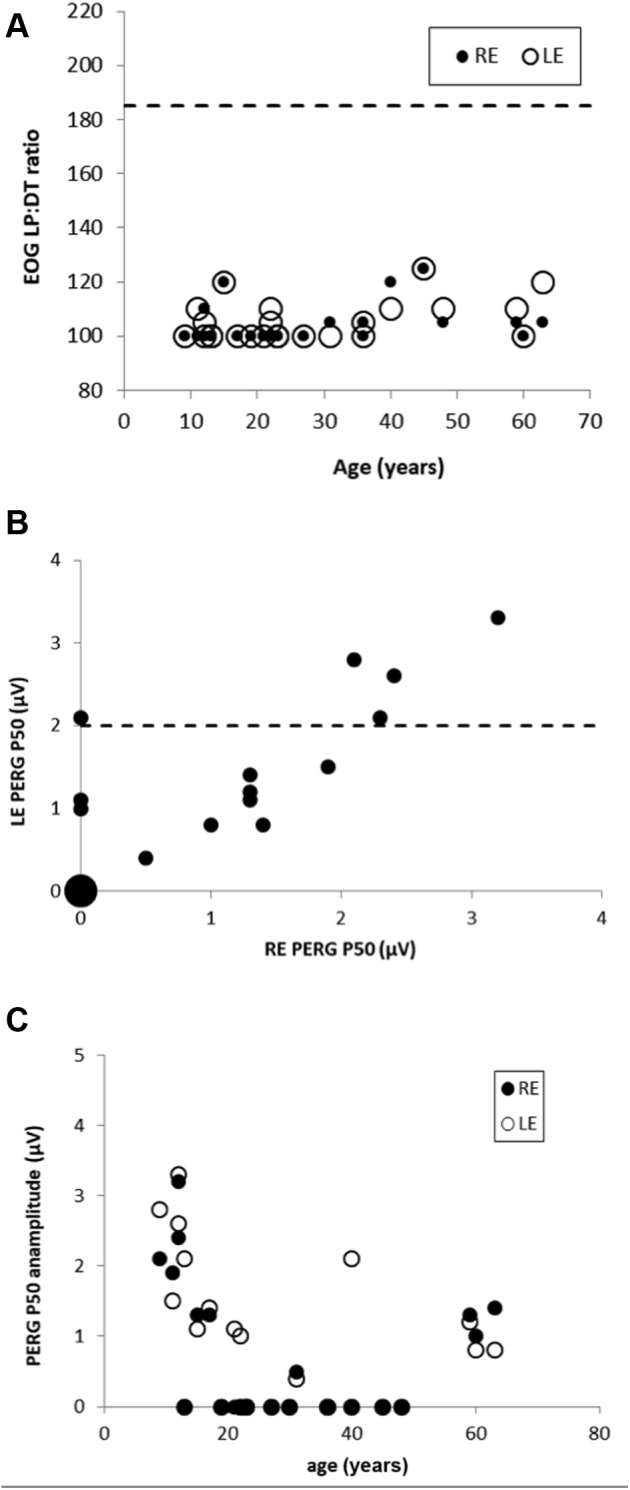
**A**, Graph showing the electro-oculogram (EOG) light peak-to-dark trough (LP:DT) ratio was grossly abnormal bilaterally (median, 100%) regardless of age. The broken line shows the lower limit of normal. **B**, Graph showing the pattern electroretinography (PERG) P50 amplitude in right eyes (RE) and left eyes (LE). The P50 component was subnormal in most eyes and undetectable bilaterally in 11 patients (large filled circle). Four patients showed an interocular asymmetry of more than 50%. The broken line shows the lower limit of normality. **C**, Graph showing the PERG findings were normal in 9 of 10 eyes, including both eyes from 4 children 9 to 13 years of age.

Electroretinography data were available for 26 patients, and electro-oculogram data were available for 24 patients. In all cases, a severe reduction in the electro-oculogram light peak-to-dark trough ratio was detected, disproportionate to the electroretinography reduction in most and in keeping with severe generalized dysfunction of the RPE. Severe electro-oculography abnormality occurred in patients of all ages and showed a high degree of interocular symmetry (Fig 6A; median light peak-to-dark trough ratio, 100%; maximum, 125%; age range, 9–63 years).

Pattern electroretinography findings were available in 51 eyes of 26 cases. Pattern electroretinography P50 was abnormal in 43 eyes, consistent with macular dysfunction, including 24 eyes from 13 patients with undetectable responses. Marked (>50%) interocular amplitude asymmetry was found in 4 patients (Fig 6B, C). Pattern electroretinography findings were normal in 9 of 10 eyes, including both eyes from 4 children 9 to 13 years of age (Fig 6C).

**Figure 7:**
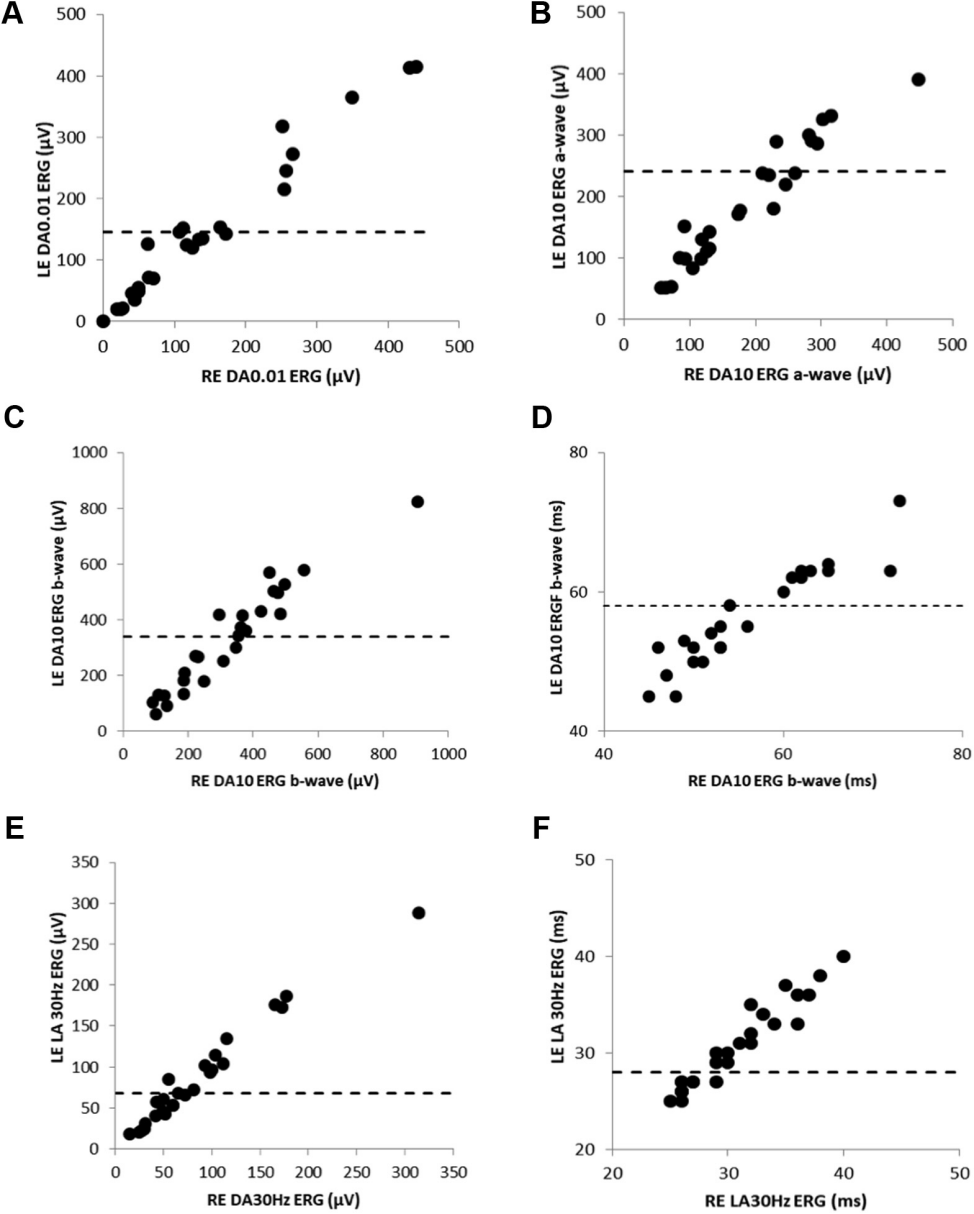
Graphs showing the main electroretinography (ERG) component amplitudes and peak times recorded from right eyes (RE) and left eyes (LE). Amplitudes are compared for the (**A**) dark-adapted (DA) 0.01 electroretinography amplitude, DA 10 electroretinography (**B**) A-wave and (**C**) B-wave amplitudes, and (**E**) light-adapted (LA) 30-Hz electroretinography amplitudes. Peak times are compared for the (**D**) DA 10 electroretinography b-wave and (**F**) LA 30-Hz flicker electroretinography amplitudes.

Full-field electroretinography findings were available in 26 patients, and the main components and interocular symmetry are summarized in Figure 7. The DA 0.01 (dim flash) and DA 10 (strong flash) electroretinography mean A- and B-wave amplitudes were 34%, 42%, and 30% smaller, respectively, than in the control group; LA 30-Hz (flicker) and LA 3 (single-flash cone) electroretinography mean amplitudes were 32% and 25% smaller, respectively, than in the control group. The mean peak time difference between patients and control participants was 6 ms for the DA 10 electroretinography B-wave amplitude and 5 ms for the LA 30-Hz electroretinography amplitude.

The DA and LA electroretinography findings indicate greater rod than cone involvement (n = 10 patients), similar severity of rod and cone system dysfunction (n = 5 patients), isolated rod dysfunction (n = 4 patients), cone more than rod dysfunction (n = 1 patient), or mild cone system involvement only (n = 2 patients). Four patients showed normal full-field electroretinography findings. Patients with normal full-field electroretinography findings were significantly younger than those with abnormal electroretinography findings (10.7 ± 3.9 years vs. 33.5 ± 16.8 years; *P* = 0.0004) and showed better VA (0.18 ± 0.13 logMAR vs. 0.57 ± 0.48 logMAR; *P* = 0.02). The main DA and LA electroretinography components showed reduction and increased peak times that tended to be worse in older patients; the mean rate of amplitude decline was similar or slightly worse than in the unaffected control group (Fig S2, available at www.aaojournal.org).

**Figure 8:**
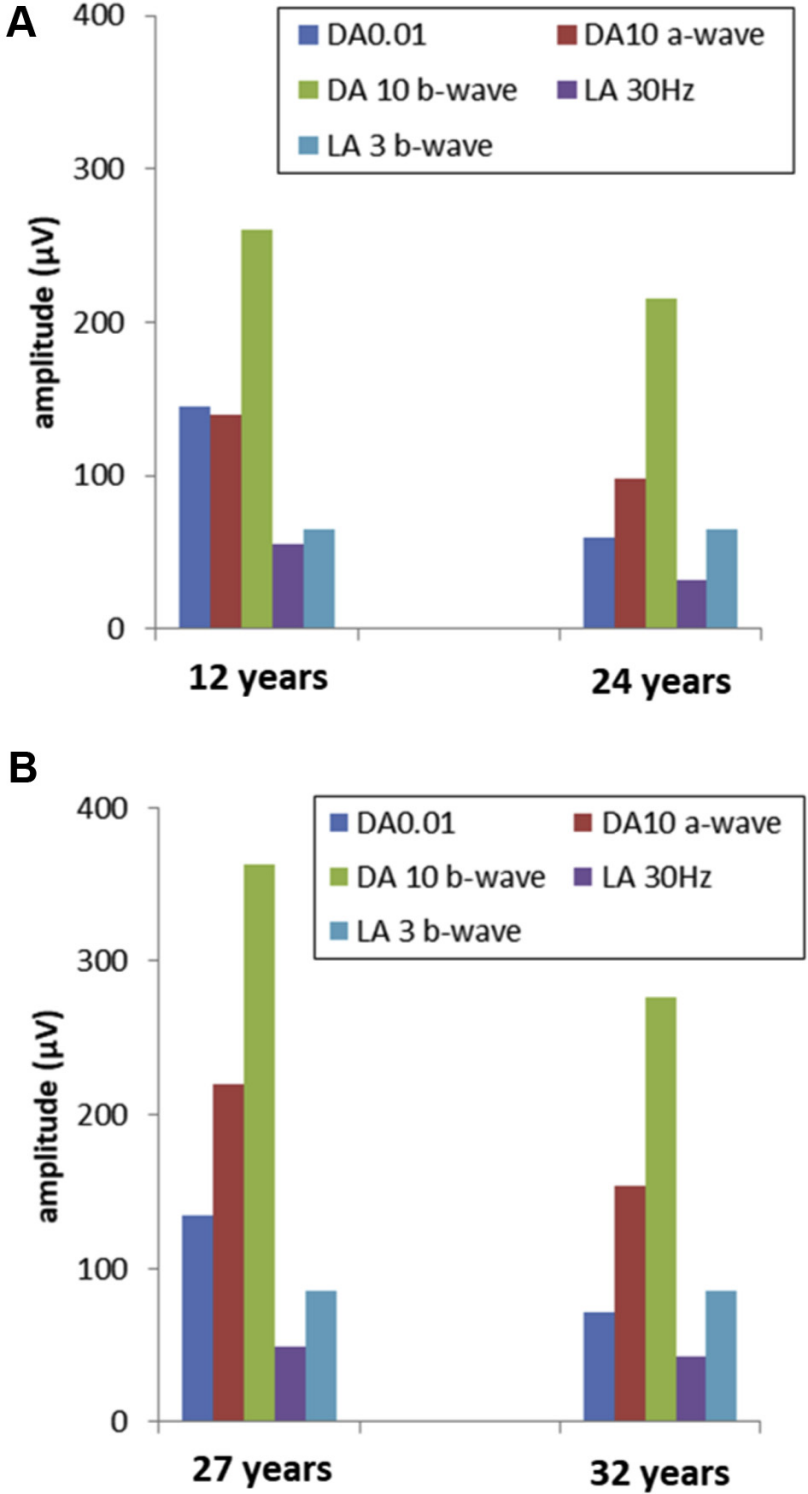
Bar graphs comparing the main electroretinography component amplitudes obtained at baseline and follow up in a (**A**) 12-year-old and (**B**) 27-year-old patient, monitored over 12 and 5 years, respectively. The light-adapted (LA) 30-Hz peak time in the younger patient increased by 8 ms after 12 years and by 7 ms in the older patient after 5 years. DA = dark-adapted.

Two patients (12 and 27 years of age at baseline) were monitored over periods of 12 and 5 years, respectively. Both showed undetectable pattern electroretinography findings. In the younger patient, DA 10 electroretinography A- and B-waves declined by 60% and 30%, respectively; LA 30-Hz electroretinography findings declined by 42% and increased in peak time by 8 ms (Fig 8A). In the older patient, DA 10 electroretinography A- and B-waves declined by 30% and 25%, respectively; LA 30-Hz electroretinography findings decreased by 12%, and peak time increased by 7 ms (Fig 8B). The rate of DA electroretinography reduction was greater, and the rate of LA electroretinography reduction was similar to that indicated by the age-dependency suggested by the cross-sectional analysis.

## Discussion

Since the recent approval of voretigene neparvovec-rzyl (Luxturna) for biallelic *RPE65*-associated retinal dystrophy, interest in gene therapy for monogenic inherited retinal dystrophies has grown. Autosomal recessive bestrophinopathy results from biallelic variants in *BEST1* and is considered the null phenotype. As such, ARB represents a possible candidate for gene replacement therapy, an idea that recently was strengthened by the promising results of *BEST1* gene supplementation in the canine model of ARB.^11^

The present work systematically reviewed the clinical and molecular features associated with ARB, representing, to the best of our knowledge, the largest series of patients to date. Unlike other early onset retinal dystrophies, children with ARB typically demonstrate good central vision, evidenced by the near-normal acuity and robust pattern electroretinography responses observed in the first decade of life. The risk of amblyopia therefore is low, as long as any associated refractive error and strabismus are corrected. Subsequently, often commencing in the teenage years, macular function declines, although this is highly variable. Although the overall trend was toward a decrease in VA over the duration of follow-up (*P* = 0.06), for the group as a whole, this did not reach statistical significance. However, in subgroup analyses, poorer VA outcomes were identified in older patients (>18 years of age vs. ≤18 years of age; *P* < 0.001) and in those with longer follow-up (≥5 years vs. <5 years; *P* = 0.001), supporting the concept of progressive deterioration. Overall, the mean rate of progression was 0.05 ± 0.11 logMAR/year, which is very similar to that observed in a recent cross-sectional cohort study of Stargardt disease (0.05 logMAR/year).^25^ A decline in visual function also was suggested by the higher prevalence of full-field electroretinography abnormalities in older compared with younger patients. Typically, these affect the rod system more than cone pathways. Where serial electroretinography assessments were performed on the same patient (n = 2), a decline of more than that expected for age was evident. Although this gradual deterioration provides a wide potential therapeutic window, this is a childhood-onset disorder, and the progression may be difficult to predict. Intervention likely is to be most effective if delivered early in the disease course and certainly before vision-limiting complications such as macular atrophy and fibrosis occur.

We also were able to identify changes in retinal structure over time, with most patients (80.4%) recording a reduction in retinal thickness during follow-up (mean follow-up, 8.6 years). Although CRT is influenced by other factors, such as the degree of IRF or SRF, the high proportion of patients recording a reduction in CRT supports the notion of progressive outer retinal atrophy. Loss of outer retinal structure may be expected to alter macular autofluorescence characteristics; herein, this was observed in 14% of patients. Macular neovascularization and FCE were 2 further independent structural changes that were identified that potentially could influence final visual prognosis. Flat irregular pigment epithelial detachments often were observed in association with FCEs, sometimes with overlying subretinal hyperreflective material; one may speculate that a neovascular lesion growing in the sub-RPE space may compromise superficial choroidal anatomic features and cause FCE more readily than a neovascular membrane that expands into the subretinal space. In addition, sub-RPE (type 1) neovascular lesions are less likely to result in dramatic, acute hemorrhagic or exudative consequences and so may be overlooked. It is interesting to note that of all monogenic retinal dystrophies, the highest prevalence of FCE is seen in association with variants in *BEST1*. In addition to the retinopathy, it is also important to remember that abnormal iridocorneal anatomic features, shallow anterior chamber depth, and reduced axial length all predispose patients to an increased prevalence of angle-closure glaucoma in those with ARB, another factor that may complicate both the delivery and response to novel therapies delivered into the vitreous or subretinal space.

Many of the imaging findings in ARB were associated with Best disease previously; however, funduscopic, retinal imaging, and electrophysiologic findings usually distinguish these two disorders.^26^ An intermediate group of patients do exist who harbor a heterozygous pathogenic *BEST1* variant associated with mild, but multifocal, subretinal deposit (multifocal Best disease).^27^ It remains to be determined if these patients truly have autosomal dominant disease, or if in fact they harbor an undetected second disease causing allele and thus represent a milder presentation of ARB. Similarly, when biallelic variants in *BEST1* are identified, there seems to be a spectrum of retinal dysfunction, with a variable age of onset of symptoms. The median age of carriers of a null allele (n = 15) was lower, 19 years, than in noncarriers (n = 12), 29 years, although the difference was not statistically significant. Furthermore, the median VA in the right eye at presentation was lower at 0.4 logMAR in null allele carriers than in noncarriers, with 0.6 logMAR VA, but was not significant. It is likely that rather than ARB representing the null phenotype, patients with these constellation of signs have significantly reduced *BEST1* function, and this may vary between no functional protein in those who are nullizygous and partial function in those with at least 1 hypomorphic, usually missense, variant associated with a milder disease with a later onset. Although null alleles may be expected to occur throughout the gene, dominantly acting variants conferring a gain of function should occur at specific residues with functional importance, as observed in autosomal dominant Best disease. Similarly, hypomorphic recessive missense variants that partially reduce *BEST1* function would be expected to cluster around in key domains; both hypotheses are supported by our data (Fig S1). A recent report by Shah et al^28^ describing a cohort of patients with *BEST1* sequence variations included 18 patients from 9 families with ARB. Missense variants were identified in all probands, in contrast to the present series, in which null alleles were discovered in 42% of patients. The most commonly identified variant in both cohorts was p.(Arg141His).

In anticipation of therapeutic trials, robust biomarkers associated with ARB disease activity are sought. Unlike many rod–cone or cone–rod dystrophies, no clear evidence exists of centrifugal or centripetal progression in patients with ARB, complicating the process of characterizing change in retinal structure. The present work suggests that conventional end points such as Early Treatment Diabetic Retinopathy Study letter score and OCT-derived measurements of retinal thickness are likely to be helpful, and although suggestions that electroretinography and FAF imaging may quantify changes in the long term (>5 years) have been noted, their usefulness in the short term (<5 years) remains to be determined. Other techniques used to assess change in visual function, such as change in electro-oculography and static perimetry, or retinal structure, such as volume of vitelliform material or fluid in the subretinal space, to date remain poorly studied in patients with ARB.

Our findings are consistent with those of other bestrophinopathies in which progressive visual worsening over time occurs, with a rate of decline that typically is slow, providing a long therapeutic window, because central photoreceptors remain viable for decades, despite the persistence of SRF.^29^ These observations are also in line with another report^26^ and support the idea that the retina may be preserved in childhood and that early treatment with gene replacement therapy may be effective in preventing later photoreceptor cell death. To date, most clinical trials of novel therapies for inherited retinal dystrophy have taken advantage of the symmetrical findings expected in these conditions. Although most potential outcome measures were found to be highly concordant between eyes (e.g., best-corrected visual acuity, CRT), in a minority of patients (4/26), the pattern electroretinography findings revealed a marked interocular difference, despite otherwise symmetrical electrophysiologic findings, and are likely to be an important consideration when considering potential treatment strategies, including potentially posing a challenge in using the fellow untreated eye as a control. Of all inherited retinal dystrophies, variants in *BEST1* seem to be associated most with unilateral or asymmetric disease.^30^ An additional factor to consider when delivering novel therapies to the macula is the association between ARB and SRF or IRF. Herein, SRF was found in the vast majority of eyes and IRF in more than one half of the eyes. Although subretinal delivery of gene replacement therapy may be less traumatic in the presence of SRF, it is likely to be more challenging if associated with IRF because of the likely greater risk of macular hole formation.^31^ Spontaneous fluctuations in IRF also are likely to impact visual function independently of any response to treatment, complicating the interpretation of visual outcome measures.

Limitations of this study that could be addressed in future work include its retrospective and predominantly cross-sectional nature and the lack of standardized protocols applied to all patients. A multicenter approach is likely to be required to increase the number of patients studied significantly, and in preparation for clinical trials, this may be possible.

In conclusion, the detailed clinical, imaging, electrophysiologic, and genetic findings of our large case series of patients with ARB will help to inform better discussions with patients regarding their prognosis and to facilitate genetic counseling and, moreover, will add to the published data to help optimize the clinical design of anticipated interventional studies, as well as providing a pool of well-characterized potential participants.

## Supplementary Data

Fig S2

Findings are separated by imaging platform as specified. Spectralis imaging was available for 56 patients and Optos imaging was available for 21 patients. The number of affected eyes is specified with the number of patients in parenthesis.
