# Supplementary material for: Autosomal Recessive Bestrophinopathy: Clinical Features, Natural History, and Genetic Findings in Preparation for Clinical Trials
Source: Ophthalmology. 2021 May;128(5):706–18. doi: 10.1016/j.ophtha.2020.10.006 (PMC8062850; doi:10.1016/j.ophtha.2020.10.006)
Supplement: Fig S1 [file mmc2.pdf]

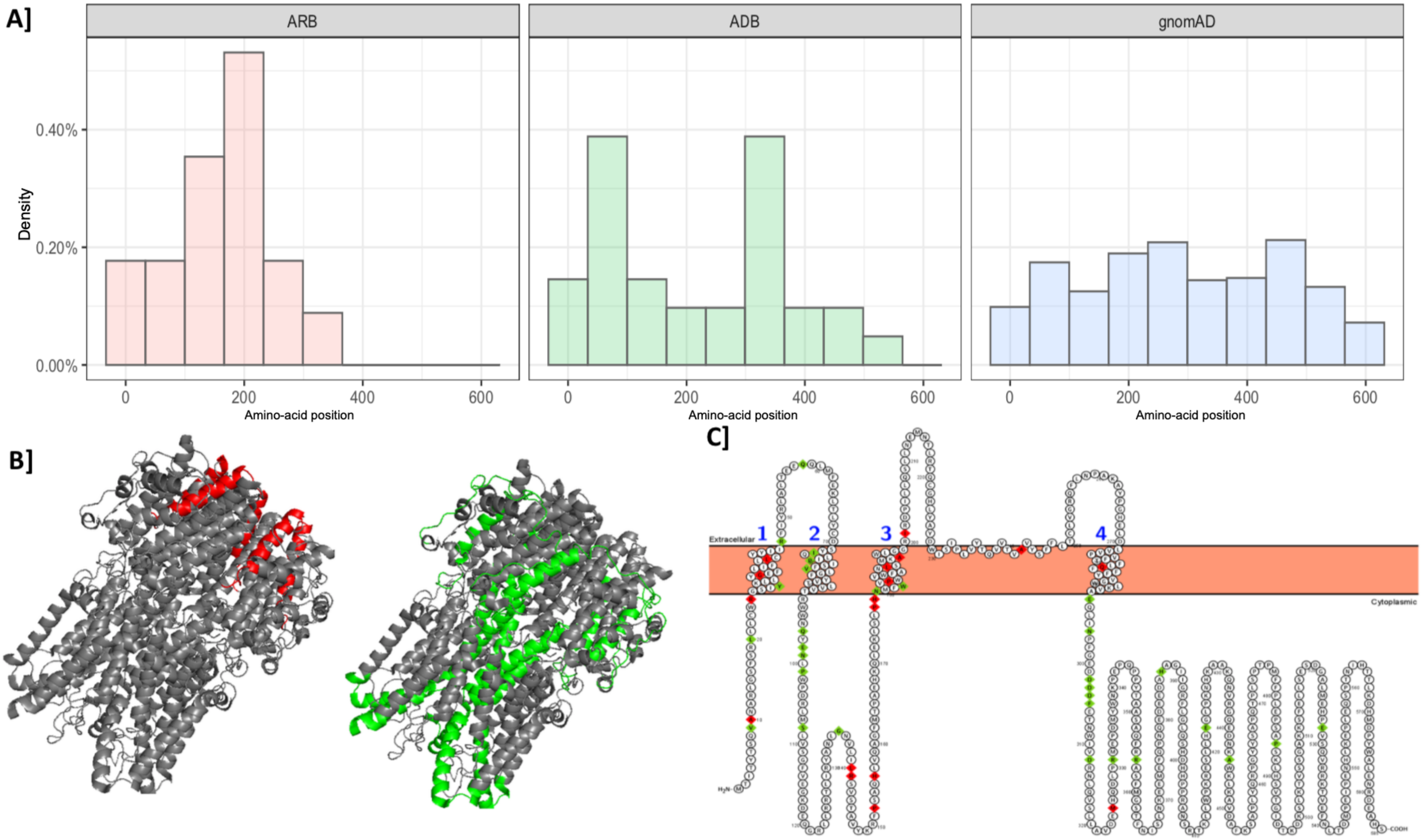

Supplemental Figure 1. Spatial clustering of missense mutations in BEST1. **A,** Histograms of the amino acid positions of missense variants in our ARB patients, (n=18), in ADB patients from Clinvar (n=31) and in controls from gnomAD (n=397) relative to ATG start codon (Met = 1). **B,** Tertiary structure of BEST1 region highlighting clustering of ARB missense variants (green) and ADB missense variants (red). **C,** Secondary structure of BEST1 with ARB missense variants (green) and ADB missense variants (red).
