# Supplementary material for: Autosomal Recessive Bestrophinopathy: Clinical Features, Natural History, and Genetic Findings in Preparation for Clinical Trials
Source: Ophthalmology. 2021 May;128(5):706–18. doi: 10.1016/j.ophtha.2020.10.006 (PMC8062850; doi:10.1016/j.ophtha.2020.10.006)
Supplement: Fig S2 [file mmc3.pdf]

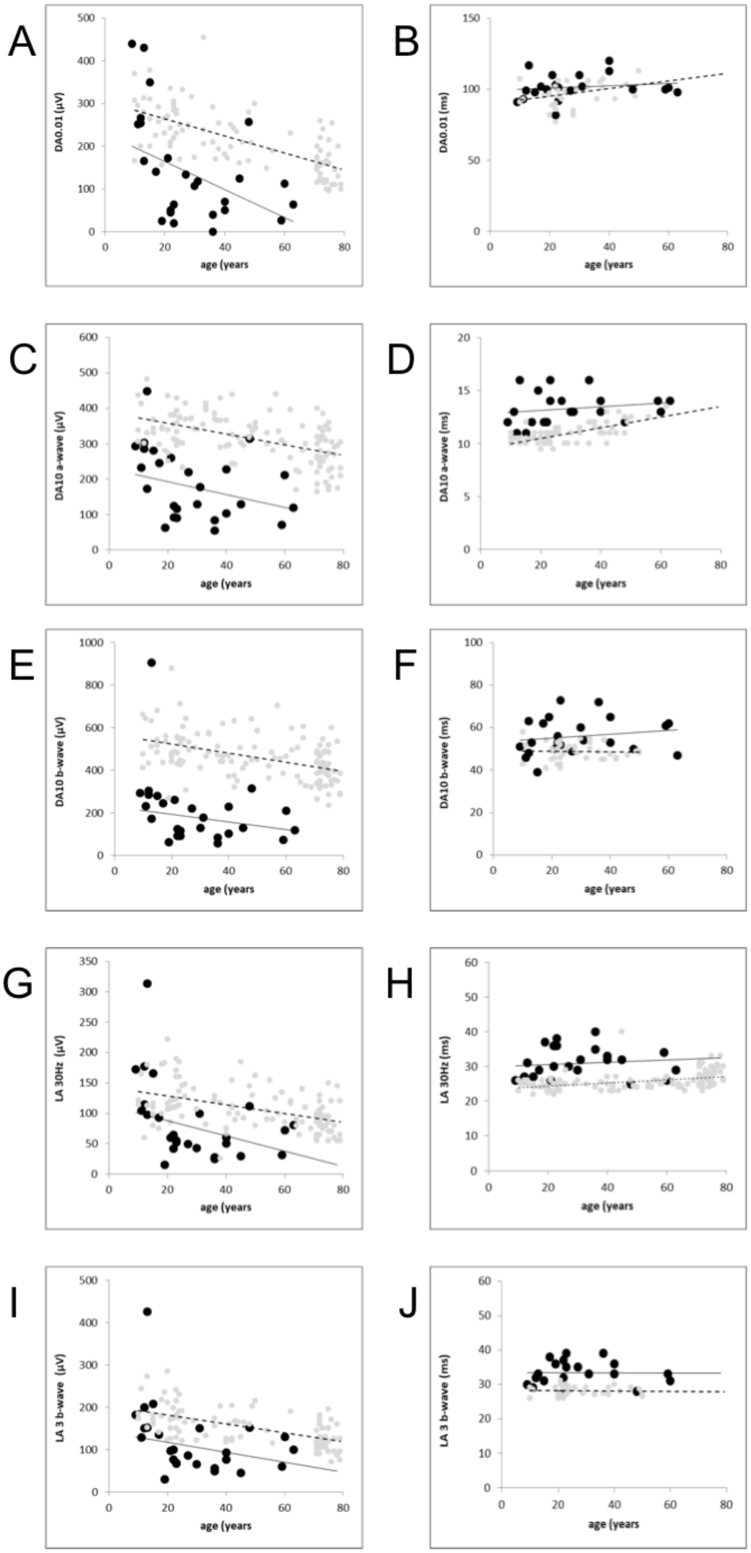

Supplemental Figure 2. The main ERG amplitude and peak times as a function of age, compared with those for an unaffected control group. Amplitude and peak time data are shown for the DA 0.01 ERG (**A, B**), the DA10 ERG a-wave (**C**, **D**) and b-wave (**E**, **F**), LA 30Hz ERG (**G, H**) and LA 3 ERG b-wave (**I**, **J**). The largest amplitude response shown in **A**, **C**, **E**, **G** and **I** (all recorded from the same young subject) are excluded from the linear regression (solid line). Linear regression for the control group is shown for comparison (broken line).
